# 870. Plasma microbial cell-free DNA sequencing impacts antimicrobial management in immunocompromised patients with pneumonia

**DOI:** 10.1093/ofid/ofad500.915

**Published:** 2023-11-27

**Authors:** Deng B Madut, Roy F Chemaly, Sanjeet S Dadwal, Joshua A Hill, Yeon Joo Lee, Ghady Haidar, Alfred Luk, Alexander Christian Drelick, Peter V Chin-Hong, Esther Benamu, Fareed Khawaja, Deepa D Nanayakkara, Genovefa Papanicolaou, Catherine Small, Monica Fung, Michelle Barron, Thomas Davis, Micah T McClain, Eileen K Maziarz, Armando Bedoya, Daniel Gilstrap, Jamie Todd, Madeleine R Heldman, Christina Barkauskaus, Robert Bigelow, Jeffrey Leimberger, Ephraim Tsalik, Olivia Wolf, Mona Mughar, Desiree Hollemon, Radha Duttagupta, Daniel Lupu, Sivan Bercovici, Bradley A Perkins, Timothy A Blauwkamp, Vance G Fowler, Thomas L Holland, Stephen P Bergin

**Affiliations:** Duke University, Durham, North Carolina; MD Anderson, Houston, Texas; City of Hope National Medical Center, Duarte, California; Fred Hutchinson Cancer Center; University of Washington, Seattle, Washington; Memorial Sloan Kettering Cancer Center, New York, New York; University of Pittsburgh School of Medicine, Pittsburg, PA; Tulane University, New Orleans, Louisiana; Weill Cornell Medicine, NewYork-Presbyterian Hospital, New York, New York; University of California San Francisco, San Francisco, California; University of Colorado, Aurora, Colorado; The University of Texas MD Anderson Cancer Center, Houston, Texas; COH, Duarte, California; Memorial Sloan Kettering Cancer Center, New York, New York; UCSF, San Fransisco, California; University of Colorado, Aurora, Colorado; Indiana University, Indianapolis, Indiana; Duke University, Durham, North Carolina; Duke University Medical Center, Durham, NC; DCRI, Durham, South Carolina; Duke University, Durham, North Carolina; DCRI, Durham, South Carolina; Duke University, Durham, North Carolina; Duke University, Durham, North Carolina; DCRI, Durham, South Carolina; Duke University, Durham, North Carolina; Duke University, Durham, North Carolina; DCRI, Durham, South Carolina; Karius, Redwood City, California; Karius, Redwood City, California; Karius Inc, Redwood City, California; Karius Inc, Redwood City, California; Karius, Inc., Redwood City, California; Karius Inc, Redwood City, California; Karius Inc, Redwood City, California; Duke University Medical Center, Durham, NC; Duke University Medical Center, Durham, NC; Duke University Health System, Durham, North Carolina

## Abstract

**Background:**

Microbial cell-free DNA (mcfDNA) sequencing can establish the etiology of multiple infectious syndromes by identifying pathogen DNA from the plasma of infected patients. Here, we describe the potential impact of a positive mcfDNA result on clinical decision making among immunocompromised adults with suspected pneumonia.

**Methods:**

This prospective observational study evaluated the potential utility of mcfDNA sequencing in adults with active hematological malignancies undergoing a diagnostic bronchoscopy for pneumonia at 10 US Medical Centers as part of the PICKUP Study (Abstract # 544 IDWeek 2022). Plasma mcfDNA was collected on all participants at the time of bronchoscopy. Clinical impact of the mcfDNA sequencing results vs usual care (UC) testing – including bronchoscopy- were adjudicated and then compared for: 1) identification of probable cause of pneumonia or clinically significant non-pulmonary infection and 2) potential changes to antimicrobial therapy if mcfDNA sequencing results were available to treating clinicians.

**Results:**

Of 223 participants analyzed, median (IQR) age was 62 (50-69) years and 72 (32.3%) were female. Plasma mcfDNA identified a probable cause of pneumonia in 57/223 (25.6%, 95% CI 20.0-31.8) participants and could have changed antimicrobial therapy in 21/57 (36.8%, 95% CI 24.4-50.7). A probable cause of pneumonia was identified by mcfDNA in 23/223 (10.3%, 95% CI 6.7-15.1) participants when no cause was identified by UC, and these detections could have resulted in an antimicrobial change in 17/23 (73.9%, 95% CI 51.6-89.8). A clinically relevant non-pulmonary infection was identified in 88/223 (39.5%, 95% CI 33.0-46.2) participants and antimicrobial therapy could have changed in 22/88 (25.0%, 95% CI 16.4-35.4). Collectively, antimicrobial therapy could have changed for 41/223 (18.4%, 95% CI 13.5-24.1) participants if mcfDNA results were available to treating clinicians.

**Figure:**
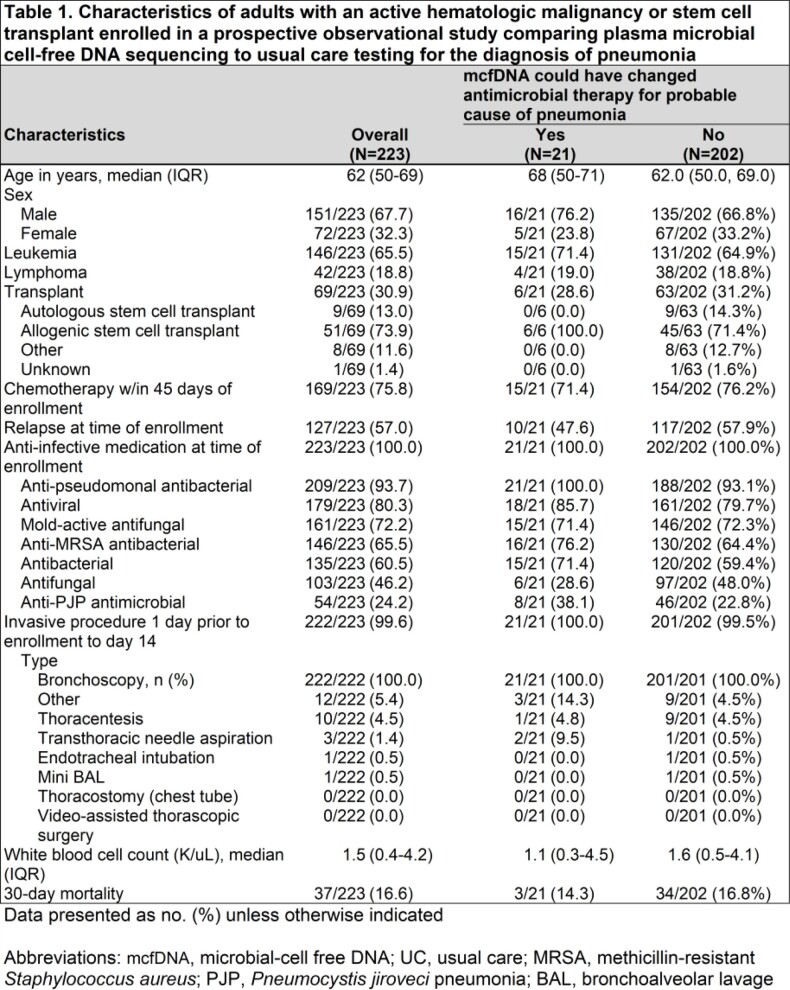

**Figure:**
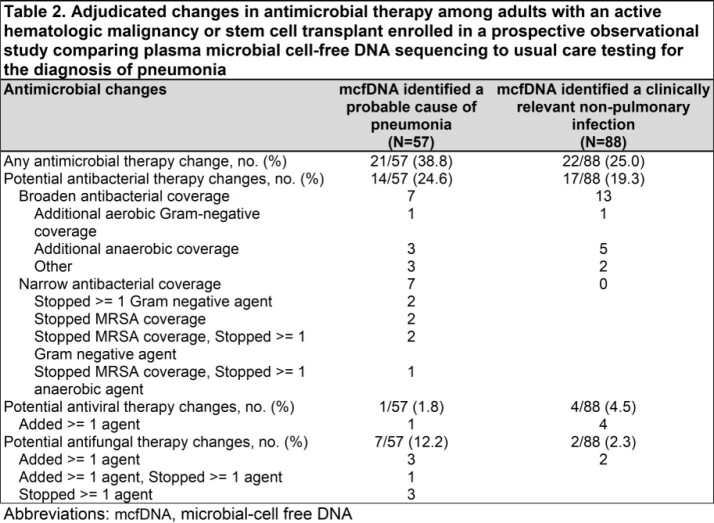

**Conclusion:**

Positive plasma mcfDNA sequencing results could have supported changes in clinical management for pneumonia and non-pulmonary infections among immunocompromised patients undergoing bronchoscopy. Further studies are needed to refine the optimal timing of mcfDNA in relation to UC testing and establish the impact of real-time mcfDNA results on patient outcomes.

**Figure:**
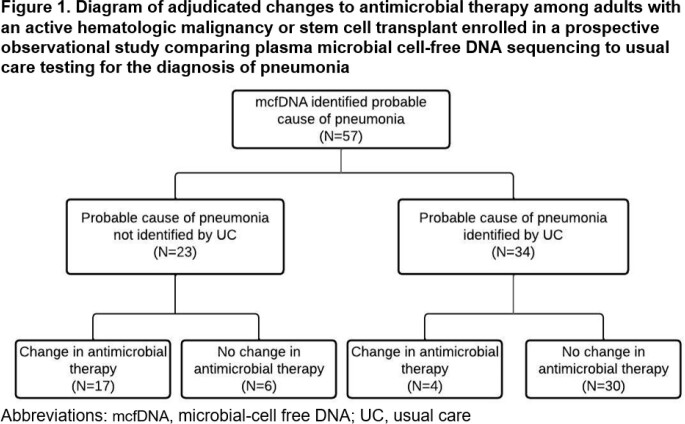

**Disclosures:**

**Deng B. Madut, MD**, Karius: Advisor/Consultant **Roy F. Chemaly, MD/MPH**, Eurofins-VViracor: Grant/Research Support|Karius: Advisor/Consultant **Sanjeet S. Dadwal, MD, FACP, FIDSA**, Allovir: Advisor/Consultant|Allovir: Grant/Research Support|Ansun Biopharma: Grant/Research Support|Aseptiscope, Inc: Stocks/Bonds|Astellas: Honoraria|Karius: Grant/Research Support|Matinas Biopharma: Stocks/Bonds|Merck: Advisor/Consultant|Merck: Grant/Research Support|Pfizer/Amplyx: Grant/Research Support|Takeda: Advisor/Consultant|Takeda: Honoraria|Viracor: Honoraria **Joshua A. Hill, MD**, Allovir: Advisor/Consultant|Allovir: Grant/Research Support|Century Therapeutics: Advisor/Consultant|Covance/CSL: Advisor/Consultant|Deverra: Grant/Research Support|Eversana Life Science Services, LLC: Advisor/Consultant|GeoVax: Grant/Research Support|Gilead: Advisor/Consultant|Gilead: Grant/Research Support|Karius: Advisor/Consultant|Karius: Grant/Research Support|Merck: Grant/Research Support|Moderna DSMB: Advisor/Consultant|Octapharma AG: Advisor/Consultant|OptumHealth: Advisor/Consultant|Oxford Immunotec: Grant/Research Support|Pfizer (previously Amplyx/Medpace): Advisor/Consultant|Senti BioSciences, Inc: Advisor/Consultant|Symbio: Advisor/Consultant|Takeda: Advisor/Consultant|Takeda: Grant/Research Support|Up-to-Date: Advisor/Consultant **Yeon Joo Lee, MD, MPH**, AiCuris: institutional research support for clinical trials|Karius: institutional research support for clinical trials|Merck: Grant/Research Support|Scynexis: institutional research support for clinical trials **Ghady Haidar, MD**, Allovir: Grant/Research Support|AstraZeneca: Advisor/Consultant|AstraZeneca: Grant/Research Support|Karius: Advisor/Consultant|Karius: Grant/Research Support|NIH: Grant/Research Support **Alfred Luk, MD**, Bill & Melinda Gates Foundation: Grant/Research Support|Karius: Advisor/Consultant|Karius: Grant/Research Support **Fareed Khawaja, MBBS**, MEDSCAPE: Honoraria|Viracor: Grant/Research Support **Genovefa Papanicolaou, MD**, Allovir: Advisor/Consultant|Amplyx: Advisor/Consultant|Astellas: Advisor/Consultant|Cidara: Advisor/Consultant|CSL Behring: Advisor/Consultant|DSMC: Advisor/Consultant|Merck: Advisor/Consultant|Merck: Grant/Research Support|Merck: institutional research support for clinical trials|MSD: Advisor/Consultant|Octapharma: Advisor/Consultant|Partners Rx: Advisor/Consultant|Shire/Takeda: institutional research support for clinical trials|Symbio: Advisor/Consultant|Symbio: Advisor/Consultant|Takeda: Advisor/Consultant|Vera Pharma: Advisor/Consultant **Micah T. McClain, MD, PhD**, Biomeme Inc: Methods to diagnose and treat acute respiratory infections **Eileen K. Maziarz, MD**, Karius, Inc: Advisor/Consultant **Robert Bigelow, PhD**, Covidien: Stocks/Bonds|Elixir Medical: Advisor/Consultant|Johnson & Johnson: Stocks/Bonds|Mckesson: Stocks/Bonds|Merck: Stocks/Bonds|Organon: Stocks/Bonds|Pfizer: Stocks/Bonds|Sanofi: Stocks/Bonds|Viatris: Stocks/Bonds **Daniel Lupu, MD, PHD**, Karius Inc: Employee|Karius Inc: Stocks/Bonds **Sivan Bercovici, PhD**, Karius: Stocks/Bonds **Bradley A. Perkins, MD**, Karius, Inc: Stocks/Bonds **Timothy A. Blauwkamp, PhD**, Karius: Board Member|Karius: Ownership Interest **Vance G. Fowler, MD, MHS**, Amphliphi Biosciences, Integrated Biotherapeutics; C3J, Armata, Valanbio; Akagera, Aridis, Roche, Astra Zeneca: Advisor/Consultant|Genentech, Regeneron, Deep Blue, Basilea, Janssen;: Grant/Research Support|Infectious Diseases Society of America: Honoraria|MedImmune, Allergan, Pfizer, Advanced Liquid Logics, Theravance, Novartis, Merck; Medical Biosurfaces; Locus; Affinergy; Contrafect; Karius;: Grant/Research Support|Novartis, Debiopharm, Genentech, Achaogen, Affinium, Medicines Co., MedImmune, Bayer, Basilea, Affinergy, Janssen, Contrafect, Regeneron, Destiny,: Advisor/Consultant|Sepsis diagnostic: Patent pending|UpToDate: Royalties|Valanbio and ArcBio: Stock Options **Thomas L. Holland, MD**, Aridis: Advisor/Consultant|Basilea Pharmaceutica: Advisor/Consultant|Karius: Advisor/Consultant|Lysovant: Advisor/Consultant **Stephen P. Bergin, MD**, Karius, Inc.: Grant/Research Support

